# Bilateral Galeazzi fracture-dislocations: a case report of early rehabilitation

**DOI:** 10.1007/s11751-012-0136-5

**Published:** 2012-05-19

**Authors:** Shingo Komura, Hidehiko Nonomura, Takashi Satake, Tatsuo Yokoi

**Affiliations:** Department of Orthopaedic Surgery, Gifu Prefectural General Medical Center, Gifu, 500-8717 Japan

**Keywords:** Galeazzi fracture-dislocations, Open reduction and internal fixation, Distal radioulnar joint, Rehabilitation

## Abstract

A 24-year-old man had bilateral Galeazzi fracture-dislocations due to a motorcycle accident. The right radius fracture was a simple fracture and was fixed with a limited contact dynamic compression plate. The left radius fracture was a comminuted fracture and was fixed with a long locking compression plate in the bridging plate fashion while maintaining reduction with a temporary external fixator. Postoperative computed tomography under passive rotation of both forearms showed acceptable congruency of the distal radioulnar joints, and early rehabilitation of forearm rotation was started at 2 weeks after the operation. At 13-month follow-up, bone union of both fractures was achieved, and forearm motion was almost restored to normal. Moreover, no subluxation or dislocation of either distal radioulnar joint was observed.

## Introduction

Galeazzi fracture-dislocation is an uncommon injury, defined as a diaphyseal fracture of the middle to distal third of the radius associated with dislocation of the distal radioulnar joint (DRUJ). The greatest problems with regard to treatment are the restoration of stability and congruency of the DRUJ. Generally, open reduction and internal fixation of radius fracture is essential in adults. However, management of the DRUJ and immobilization of the forearm are still controversial [1–4]. Despite prolonged immobilization, instability or subluxation of the DRUJ may occur, and it is therefore difficult to determine the appropriate duration of immobilization and the best timing for beginning rehabilitation. Here, we report a rare case of bilateral Galeazzi fracture-dislocations and early rehabilitation for this injury.

## Case report

**Fig. 1:**
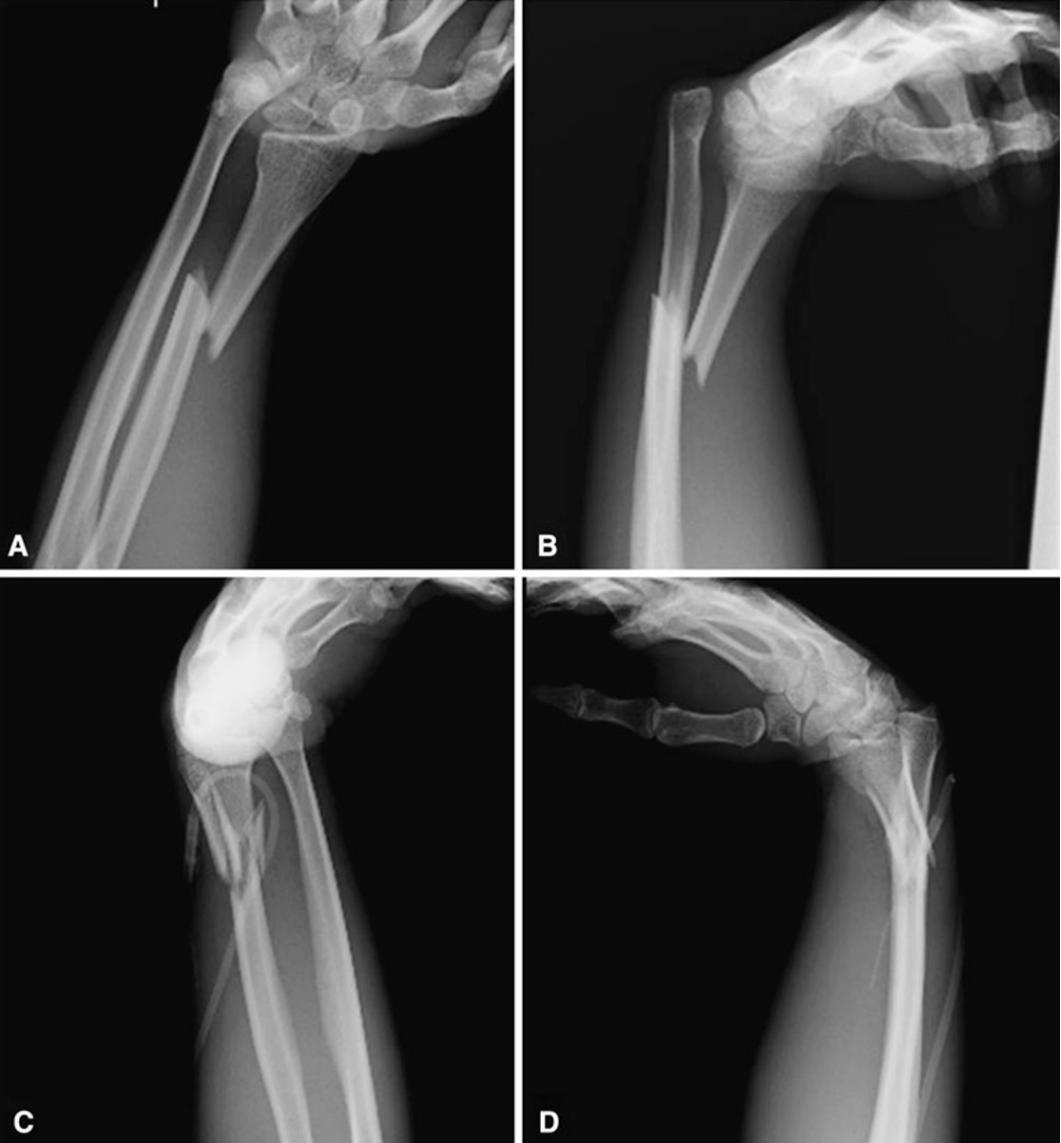
Anteroposterior and lateral radiographs of both forearms at initial diagnosis. Simple fracture of the *right* radius (**a**, **b**) and comminuted fracture of the *left* radius (**c**, **d**) were observed. Both distal ulnar heads were dislocated dorsally

**Fig. 2:**
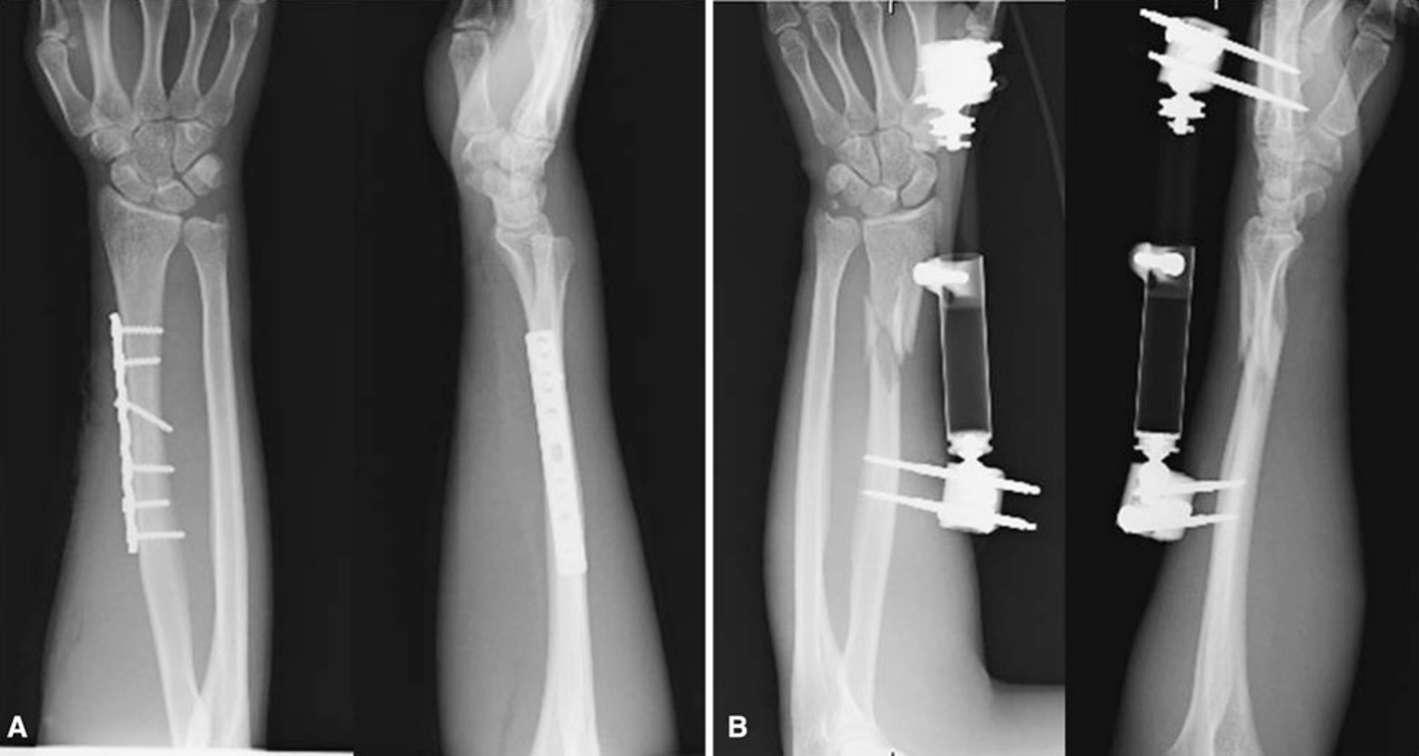
Anteroposterior and lateral radiographs after emergency operation. Despite anatomical reduction in the *right* radius, dorsal subluxation of the ulnar head was observed. The *left* radius was fixed with an external fixator, and DRUJ was reduced. Distal pins were inserted into the second metacarpal bone and proximal pins into the radius shaft

**Fig. 3:**
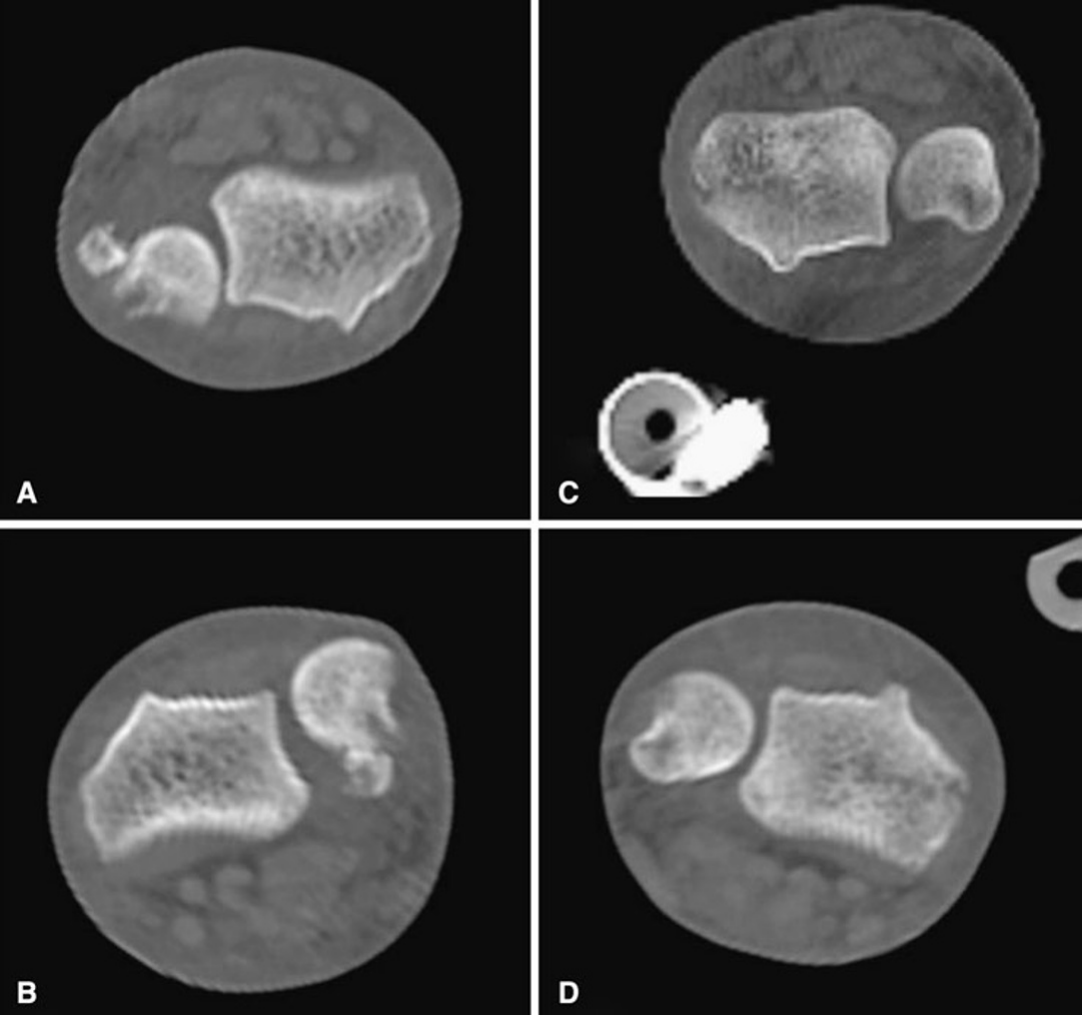
Axial plane CT imaging after emergency surgery. **a***Right* in supination. **b***Right* in pronation. **c***Left* in supination. **d***Left* in pronation. Persistent dorsal subluxation of the *right* ulnar head was observed (**a**, **b**), whereas the left DRUJ showed almost normal congruity (**c**, **d**)

**Fig. 4:**
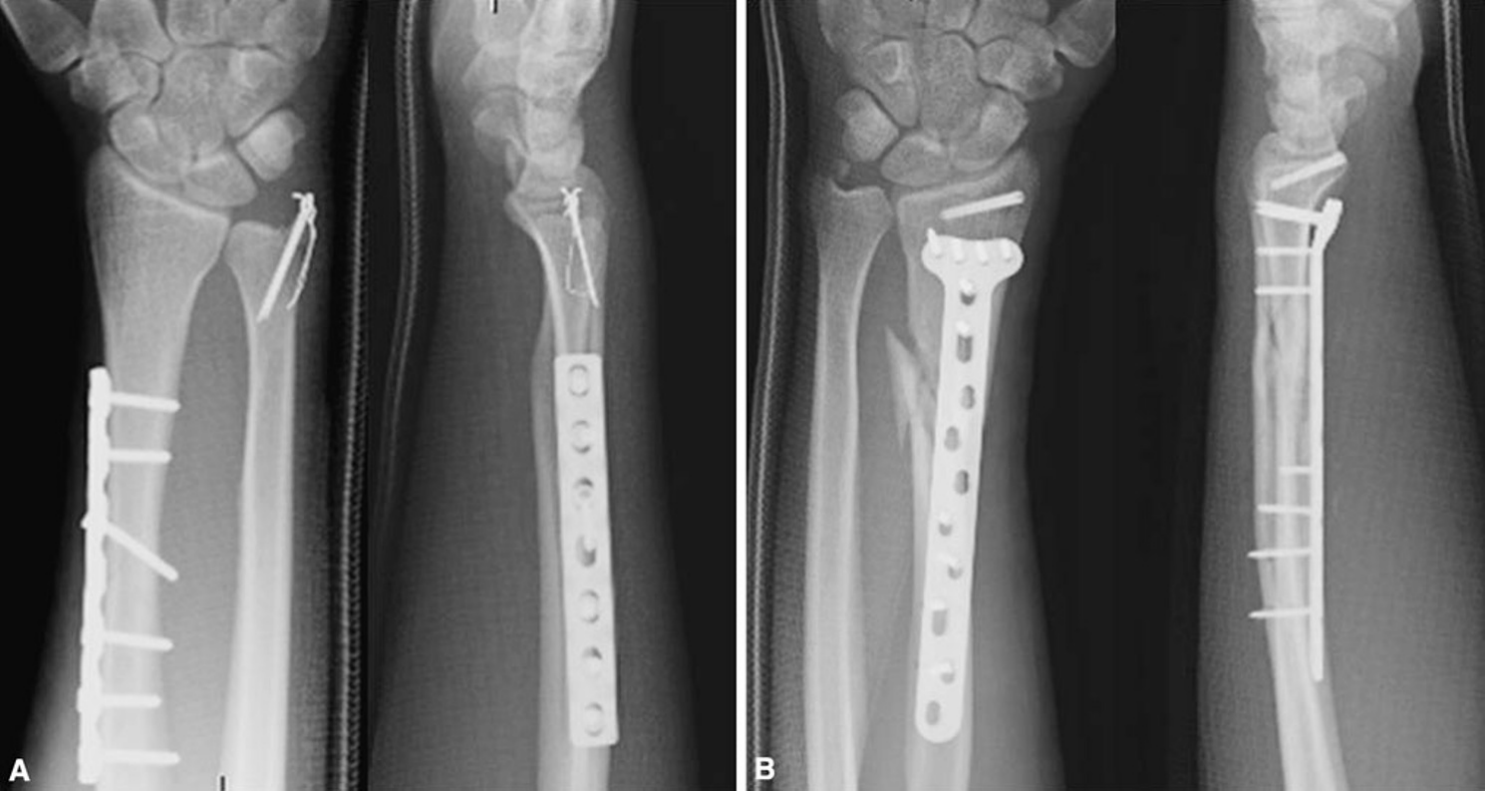
Anteroposterior and lateral radiographs after additional surgery. **a***Right*. **b***Left*

**Fig. 5:**
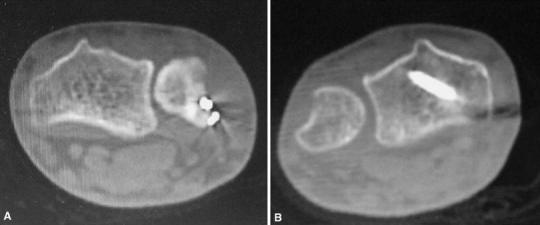
Axial plane CT imaging after surgery. **a***Right* in pronation. **b***Left* in pronation. Both DRUJs showed almost normal congruity

**Fig. 6:**
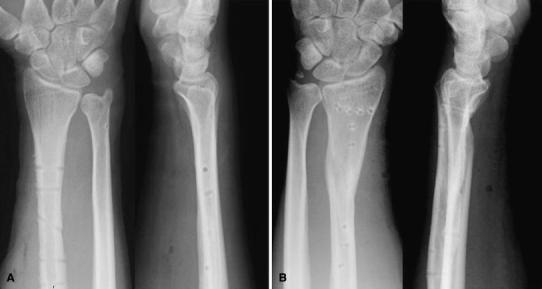
Anteroposterior and lateral radiographs at 13-month follow-up. **a***Right*. **b***Left*. Bone union of both forearms was achieved, and neither subluxation nor dislocation of the distal ulnar heads was observed

A 24-year-old man was riding a motorcycle and collided with a large truck crossing the street ahead him. Radiography revealed bilateral Galeazzi fracture-dislocations with dorsally dislocated ulnar heads and ulnar styloid fractures (Fig. 1). Closed reduction was performed under sedation, but both fracture-dislocations were irreducible. Three hours after the injury, the patient underwent an emergency operation. The right simple radius fracture was fixed with a 7-hole 3.5-mm limited contact dynamic compression plate (LC-DCP; Synthes, Oberdorf, Switzerland) through an anterior approach. Due to the lack of a suitable implant, the left comminuted radius fracture was fixed temporarily with an external fixator, to reduce the dislocated ulnar head. However, the patient complained of pain in his right wrist, and postoperative radiography of the right side revealed dorsal subluxation of the ulnar head (Fig. 2). Computed tomography (CT) performed under passive pronation and supination showed acceptable congruity of the left DRUJ in rotation, along with persistent dorsal subluxation of the right ulnar head (Fig. 3). One week after the primary operation, the patient underwent an additional operation. The left comminuted radius fracture was fixed with a locking compression plate (LCP), consisting of an extra-long 10-hole 2.4-mm distal radius plate (Synthes) in bridging plate fashion through the anterior approach while maintaining reduction with an external fixator. Iliac cancellous bone was grafted due to improved fracture healing. Following achievement of stable fixation of the radius with the LCP, the external fixator was removed. A manual stressing test under fluoroscopic imaging showed that the left DRUJ was slightly loose, but its congruity was good, and passive forearm rotation was smooth and fully possible, thereby obviating the need for additional internal fixation of the ulnar styloid. To reduce the dorsally dislocated ulnar head, the right DRUJ was exposed on the ulnar head through a dorsoulnar approach. There were no tears in the triangular fibrocartilage complex (TFCC) and no tendon incarceration in the DRUJ. We observed palmar displacement of the avulsion fragment of the ulnar styloid process by the ulnar collateral ligament and periosteum of the ulna, with the avulsion fragment causing dorsal displacement of the ulnar head. The fragment was fixed by tension band wiring fixation, resulting in the anatomical reduction and stabilization of the DRUJ. Both arms were splinted in supination (Fig. 4). Immediately, postoperative rehabilitation of the wrist and elbow was started without restriction, and active and active-assisted rotation of the forearm from neutral to full supination were allowed. Two weeks after the 2nd operation, CT under gentle passive rotation was reexamined. Acceptable reduction of the distal ulnar head and stability of both DRUJs in pronation were confirmed (Fig. 5). and active rotation of both forearms was started. Splints were removed at 5 weeks, and passive rotation was permitted. Nine months after the injury, all hardware of both forearm were removed. At the 13-month follow-up, radiographs showed no subluxation of both ulnar heads (Fig. 6). The patient’s forearm motion was restored to almost normal, with pronation and supination of 75° and 85° on the right and 80° and 85° on the left, respectively. Both DRUJs were slightly loose, and the patients had mild pain in his right wrist in hyperpronation; however, the patient had no pain in his left wrist and returned to normal life as a factory worker.

## Discussion

**Fig. 7:**
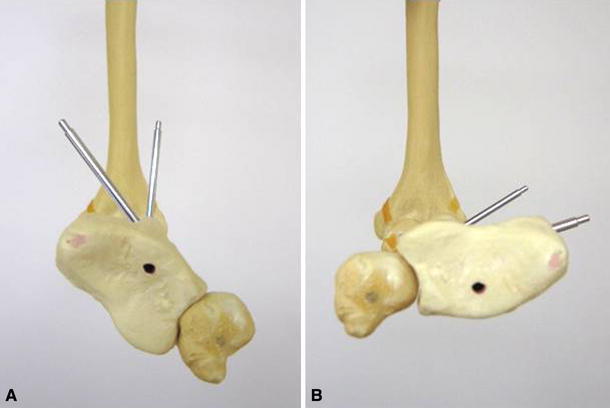
Axial view of the normal DRUJ in the left wrist (bone model). **a** Pronation. **b** Supination

Traditionally, immobilization of the forearm in full supination for between 6 and 8 weeks has been recommended in Galeazzi fracture-dislocations [4]. Adams recommended that DRUJ dislocations be treated by long-arm casting in a stable position of forearm rotation for 3–4 weeks followed by use of a well-molded short-arm cast for 2–3 weeks [5], and Giannoulis et al. [6] recommended long-arm cast immobilization for 4–6 weeks after appropriate stabilization of the DRUJ as follows: when the DURJ is stable, the DRUJ is fixed with 2 Kirschner wires in neutral or mild supination; when the DRUJ is unstable, the triangular fibrocartilage complex is repaired or ulnar styloid fracture is fixed. Prolonged immobilization is still recommended. However, postoperative treatment for Galeazzi fracture-dislocation may be open to further discussion depending on the congruity and stability of the DRUJ. Recently, Gwinn et al. [7] reported early motion protocol for Galeazzi fracture-dislocation, and 92% of the patients successfully completed the protocol (Table 1). This report suggests that early forearm rotation of most Galeazzi fracture-dislocations was possible following acceptable reduction and stability of both the radius and the DRUJ. Accurate posteroanterior and lateral radiographs and physical examination, including forearm rotation and manual stressing tests under fluoroscopic imaging, are generally required to assess the reduction and stability of the DRUJ [7]. However, we believe that the congruity of the DRUJ should be assessed during forearm rotation (Fig. 7). We hypothesized that if congruity was maintained throughout the full range of forearm rotation, subluxation or redislocation of the DRUJ would not occur, even early during the rehabilitation of forearm rotation. We therefore attempted the early mobilization of Galeazzi fracture-dislocations, with evaluation by CT imaging during forearm rotation. The congruity of DRUJ during forearm rotation was confirmed, and early rotation of the forearms was begun. Despite early rehabilitation of forearm rotation 2 weeks after the operation, no resubluxation or redislocation of the DRUJs occurred, and the range of motion of the forearms was restored to almost normal.

**Table 1:** Postoperative rehabilitation protocol for Galeazzi fracture-dislocation according to Gwinn et al. [7]

1. First 2 weeks: Wrist immobilization in 30 degrees of supination with the elbow free fully flex and extend in orthotic or cast
2. Next 2 weeks: Active and active-assisted rotation of the wrist from neutral to supination
3. Next 2 weeks: Full wrist range motion in all planes with both active and active-assisted range of motion
4. At 6 weeks: Initiation of strengthening exercise and no further limitation to wrist motion

Typically, in Galeazzi fracture-dislocation, a dorsal dislocation of the ulnar head is most stable in supination and a palmar dislocation in pronation [5]. Supination of the forearm can provide good congruity of the DRUJ, whereas pronation might result in dorsal dislocation of the ulnar head. Since the first CT examination and radiographs after additional surgery showed that both DRUJs were stable palmarly, we assessed both DRUJs only in pronation during the second CT examination. An additional examination in supination may have resulted in a more accurate assessment of the congruity of both DRUJs, establishing the usefulness of CT evaluation in determining the start of rehabilitation more safely.

Our findings suggest that in patients with well-reduced and fixed Galeazzi fracture-dislocation, the postoperative congruity of the DRUJ should be evaluated by CT imaging during passive forearm rotation. Although both active and passive rotation can be used to evaluate the congruity of the DRUJ, patients with postoperative pain in the wrist may be unable to rotate their forearm sufficiently. To assess whether the congruity of the DRUJ is sufficient, CT images of the wrist should be obtained in maximum supination and pronation, making passive rotation of the forearm more useful. This method may be useful in deciding when to start early rehabilitation of forearm rotation.
